# Celastrol prevents high‐fat diet‐induced obesity by promoting white adipose tissue browning

**DOI:** 10.1002/ctm2.641

**Published:** 2021-12-15

**Authors:** Bingwei Wang, Xiaoning Yang, Miao Zhao, Zhijie Su, Zhiping Hu, Chenyu Zhang, Bingbing Guo, Jiarui Liu, Lihua Qin, Weiguang Zhang, Ruimao Zheng

**Affiliations:** ^1^ Department of Anatomy Histology and Embryology School of Basic Medical Sciences Health Science Center Peking University Beijing China; ^2^ Department of Hepatobiliary Surgery, Peking University People's Hospital Peking University Beijing China; ^3^ Neuroscience Research Institute Peking University Beijing China; ^4^ Key Laboratory for Neuroscience of Ministry of Education Peking University Beijing China; ^5^ Key Laboratory for Neuroscience of National Health Commission Peking University Beijing China

**Keywords:** celastrol, obesity, white adipose tissue browning, fatty acid transport 2

Dear Editor,

Despite advances in therapy for obesity, effective therapeutic strategies for weight loss are still needed.^1^ Our team revealed that celastrol promoted white adipose tissue (WAT) browning, and protected against high‐fat diet (HFD)‐induced obesity via activation of hypothalamus ‐ sympathetic nervous system ‐ β_3_AR axis. Fatty acid transport 2 (Fatp2) may serve as an important factor to mediate the browning‐inducing effects of celastrol. This study may provide a basis for exploring a new strategy for the treatment of obesity.

Celastrol is a leptin sensitizer.^2^ We found that long‐term treatment with celastrol at doses of 10 and 1 μg/kg prevented the development of obesity (Figure S1A,B). Food intake was suppressed by celastrol at dosages of 1000, 100 and 10 μg/kg, but not 1 μg/kg (Figure S1C,D). Celastrol treatment at doses of 1000 and 100 μg/kg resulted in a severe decline in food intake and body weight in HFD‐fed mice by 4–5 days (Figure S1A–C). Increased UCP1 expression was observed in inguinal WAT (iWAT) of the mice treated with celastrol at a dose of 1 μg/kg (Figure S1E). The canonical molecular and morphological characteristics of WAT browning including the increased protein levels of uncoupling protein‐1 (UCP1) and peroxisome proliferator‐activated receptor‐UCP1 and PGC1γ coactivator 1‐α (PGC1α), and the decreased size of multilocular adipocytes were also seen in iWAT (Figure S1F–H). The increased expressions of WAT browning‐associated genes were not seen in mice treated with celastrol at a dose of .1 μg/kg (Figure S1I). The HFD‐induced obesity was prevented by celastrol at the dose of 1 μg/kg during a 3‐month treatment without affecting food intake (Figure 1A–E). A sustained expression of WAT browning‐associated genes was observed in iWAT of celastrol‐treated mice (Figure 1F). Celastrol diminished the increment of adiposity in both room temperature (RT) and thermoneutrality (Figure 1G–I,K). No significant difference in the food intake was found (Figure 1J). Celastrol stimulated expressions of WAT browning‐associated genes in iWAT at RT and thermoneutrality (Figure 1L), which was accompanied by increased protein levels of UCP1 and PGC1α and multilocular beige adipocytes (Figure 1M–O). Celastrol increased the levels of several long‐chain polyunsaturated fatty acids (Figure 1P). Celastrol increased energy expenditure (EE) and decreased respiratory exchange ratio (RER) without affecting motor activity (Figure 1Q–S).

**FIGURE 1:**
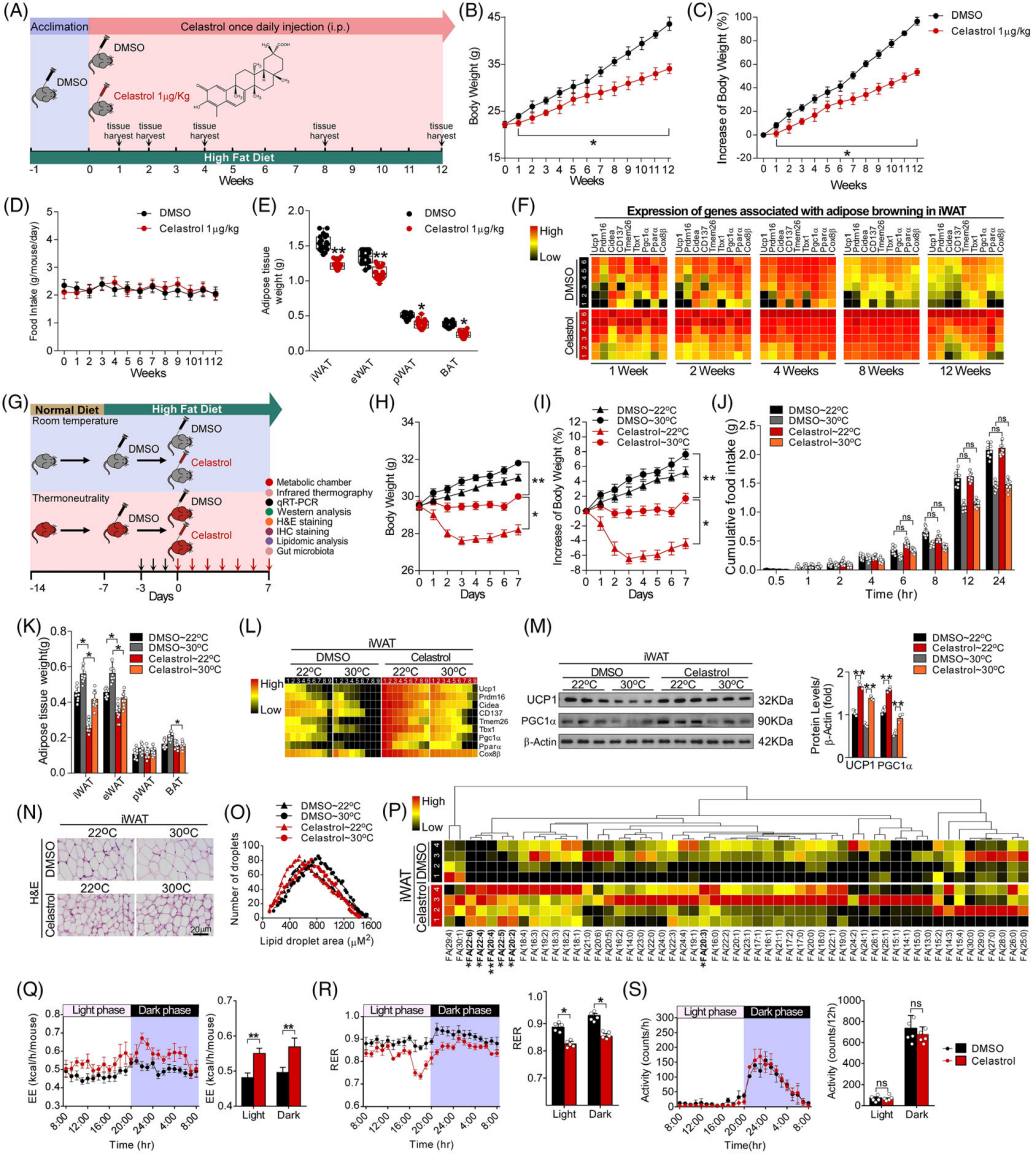
Celastrol prevents high‐fat diet (HFD)‐induced obesity and promotes white adipose tissue (WAT) browning. (A) Diagram of the experimental design. Celastrol (1 μg/kg) or dimethyl sulfoxide (DMSO) was injected (i.p.) daily for 12 consecutive weeks starting on week 0 after acclimation, then tissues were harvested for molecular analyses at week 12 after the last administration. (B) Body weight (*n* = 20 per group). (C) Percentage change in body weight (*n* = 20 per group). (D) Food intake (*n* = 20 per group). (E) Fat‐pad weight (*n* = 20 per group). Epididymal WAT (eWAT), retroperitoneal WAT (pWAT) and brown adipose tissue (BAT). (F) Heatmap shows mRNA levels of browning associated genes in inguinal WAT (iWAT) of Celastrol or DMSO treated mice (*n* = 6 per group). Values represent mean ± standard error of the mean (SEM). *p*‐Values were determined by non‐paired two‐tailed Student's *t*‐test. *p < .05. (G) Diagram of the experimental design. Celastrol (1 μg/kg) or DMSO was injected (i.p.) daily for 7 consecutive days starting on day 0 after acclimation (3 days), then indirect calorimetry recording was performed on day 7 and tissues were harvested for molecular analyses on day 8. The arrow indicates the time of Celastrol or DMSO injection. (H) Body weight (*n* = 9 per group). (I) Percentage change in body weight (*n* = 9 per group). (J) Cumulative food intake (*n* = 9 per group). (K) Fat‐pad weight (*n* = 9 per group). (L) Heatmap shows mRNA levels of browning associated genes in iWAT (*n* = 9 per group). (M) Representative immunoblots of UCP1, PGC1α and β‐Actin from iWAT, and the quantified ratio of UCP1/β‐Actin, PGC1α/β‐Actin (*n* = 9 per group). Cropped blot images are shown, see Figure S5 for full immunoblots. (N) Representative images of H&E staining of iWAT (*n* = 9 per group). Scale bar indicates 20 μm. (O) The cell size profiling of adipocytes from iWAT and the quantitative analysis by ImageJ program (*n* = 9 per group). Values represent mean ± SEM. *p*‐ Values were determined by two‐way analysis of variance (ANOVA) followed by Tukey's multiple comparisons test. *p < .05, **p < .01. (P) Lipidomics heatmap showing the average changes in free fatty acids of iWAT (*n* = 4 per group). (Q) Energy expenditure. Analysis of covariance (ANCOVA) was used to determine statistical differences for in vivo metabolic analyses. (R) Respiratory exchange ratio (RER). (S) Physical activity (*n* = 5 per group). Values represent mean ± SEM. *p*‐Values were determined by non‐paired two‐tailed Student's *t*‐test. *p < 0.05.

Hypothalamus controls sympathetic activity, which is involved in the regulation of WAT browning (Figure S2A). Tyrosine hydroxylase (TH), a marker of sympathetic nervous activity (SNA), was increased in iWAT of celastrol‐receiving mice (Figure S2B,C). Gene set enrichment analysis (GSEA) demonstrated that SNA was upregulated in celastrol‐treated iWAT (Figure S2D). The deficiency of β_3_‐adrenergic receptor (AR) abrogated the weight‐reducing effects of celastrol without affecting food intake (Figure 2A–D), and β_3_AR‐knockout (KO) diminished the expression of WAT browning‐associated genes (Figure 2E). Western analysis revealed a similar change in TH, UCP1 and PGC1α (Figure 2F). The larger cell size and cytoplasmic unilocular lipid droplets were seen in the iWAT of Adrb3‐KO mice (Figure 2G,H). Similar results were seen in the mice treated with β‐blockers (Figure S3A–H).

**FIGURE 2:**
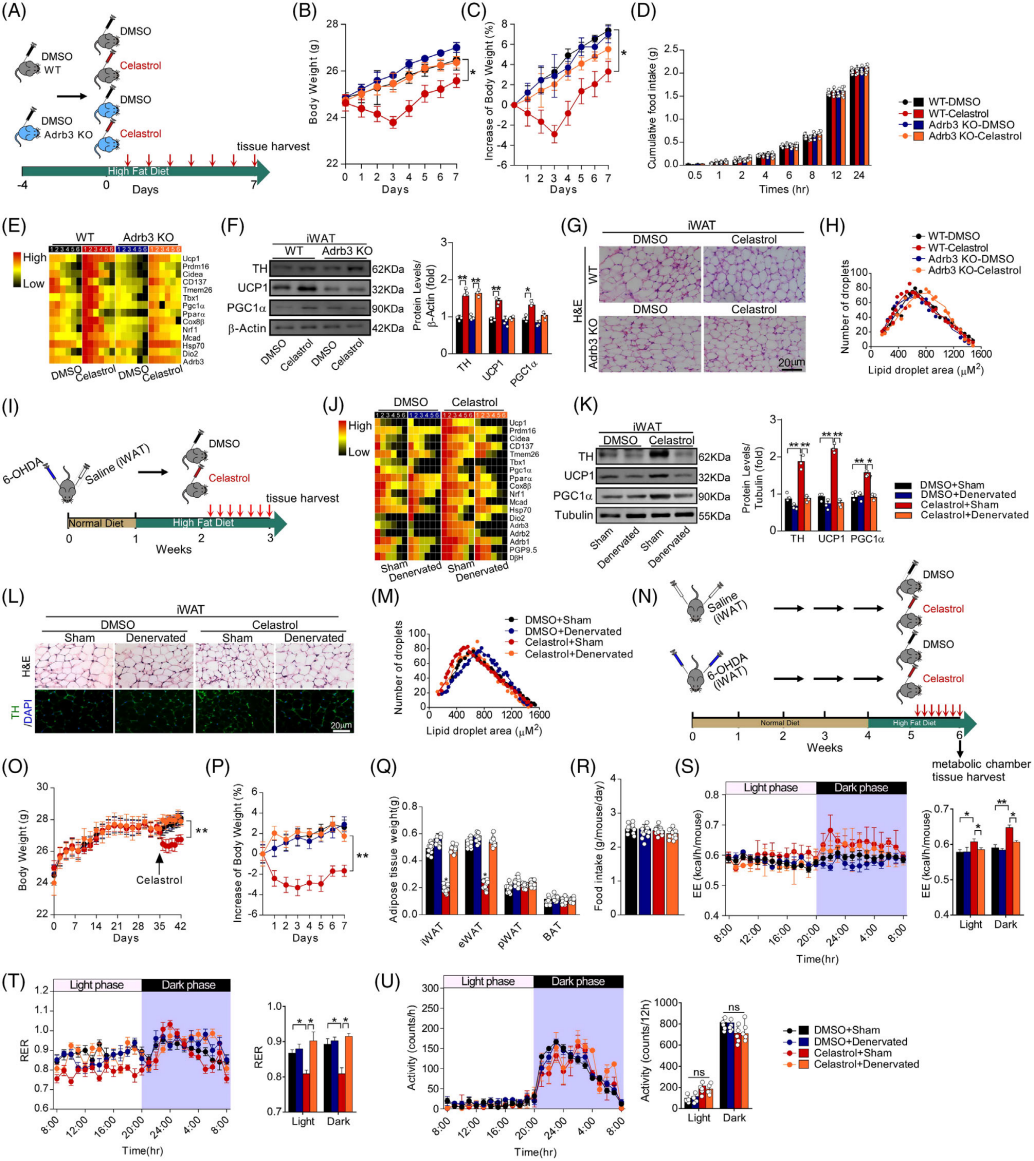
Celastrol promotes white adipose tissue (WAT) browning through the sympathetic nervous system (SNS)‐Adrb3 axis. (A) Schematic illustration of experiments. Mice received Celastrol or DMSO once daily for 7 consecutive days after 4‐day acclimation. Mice were kept on high‐fat diet (HFD). Tissues were harvested for molecular analyses on day 8 after the last administration. (B) Body weight (*n* = 9 per group). (C) Percentage change in body weight (*n* = 9 per group). (D) Cumulative food intake (*n* = 6 per group). (E) Heatmap shows mRNA levels of browning associated genes in inguinal WAT (iWAT) (*n* = 6 per group). (F) Representative immunoblots of tyrosine hydroxylase (TH), UCP1, PGC1α and β‐Actin from iWAT, and the quantified ratio of TH/β‐Actin, UCP1/β‐Actin, PGC1α/β‐Actin (*n* = 9 per group). Cropped blot images are shown, see Figure S5 for full immunoblots. Values represent mean ± standard error of the mean (SEM). *p*‐Values were determined by two‐way analysis of variance (ANOVA) followed by Tukey's multiple comparisons test. *p < .05, **p < .01. (G) Representative images of H&E staining of iWAT (*n* = 7–9 per group). Scale bar indicates 20 μm. (H) The cell size profiling of adipocytes from iWAT and the quantitative analysis by ImageJ program (*n* = 8 per group). (I) Schematic illustration of experiments. iWAT was unilaterally denervated with 6‐hydroxydopamine (6‐OHDA), 2 weeks later, mice received Celastrol or dimethyl sulfoxide (DMSO) once daily for 7 consecutive days. Mice were kept on HFD for 2 weeks. Tissues were harvested for molecular analyses at week 3. (J) Heatmap shows mRNA levels of browning associated genes in iWAT (*n* = 10 per group). (K) Representative immunoblots of TH, UCP1, PGC1α and β‐Actin from iWAT, and the quantified ratio of TH/β‐Actin, UCP1/β‐Actin, PGC1α/β‐Actin (*n* = 10 per group). Cropped blot images are shown, see Figure S5 for full immunoblots. Values represent mean ± SEM. *p‐*Values were determined by two‐way ANOVA followed by Tukey's multiple comparisons test. *p < .05, **p < .01. (L) Representative images of H&E staining and immunofluorescence images of TH in iWAT (*n* = 10 per group). Scale bar indicates 20 μm. (M) The cell size profiling of adipocytes from iWAT and the quantitative analysis by ImageJ program (*n* = 10 per group). (N) Schematic illustration of experiments. iWAT was bilaterally denervated with 6‐OHDA, 5 weeks later, mice received celastrol for 7 consecutive days. Mice were kept on HFD for 2 weeks. Indirect calorimetry was performed and tissues were harvested for molecular analyses at week 6. (O) Body weight (*n* = 10 per group). (P) Percentage change in body weight (*n* = 10 per group). (Q) Fat‐pad weight (*n* = 10 per group). (R) Food intake (*n* = 10 per group). *p*‐Values were determined by two‐way ANOVA followed by Tukey's multiple comparisons test. *p < .05. (S) Energy expenditure. Analysis of covariance (ANCOVA) was used to determine statistical differences for in vivo metabolic analyses. (T) Respiratory exchange ratio (RER). (U) Ambulatory physical activity (*n* = 5 per group). Values represent mean ± SEM. *p*‐Values were determined by non‐paired two‐tailed Student's *t*‐test. *p < .05

As reported,^3^ we denervated sympathetic nerves in iWAT. The unilateral sympathetic denervation diminished the raised expression of WAT browning‐associated genes (Figure 2I–K). The denervated iWAT showed larger cell size and unilocular lipid droplets (Figure 2L,M). The iWAT bilateral sympathetic denervation dampened the weight‐reducing effects without affecting food intake and ambulatory activity (Figure 2N–R,U). A decreased EE was observed in the denervated celastrol‐treated mice compared with controls (Figure 2S,T). Taken together, these findings indicate that sympathetic nerves mediate the WAT browning process induced by celastrol.

Furthermore, we found that the increased expression of WAT browning‐associated factors was normalized after denervation (Figure 3A). The genes related to catabolic metabolism were increased, while the genesassociated with inflammation were decreased in the iWAT of celastrol‐treated mice (Figure 3B,C). The expression of genes associated with WAT browning, fat mobilization, glucose metabolism and SNA were upregulated in the celastrol plus sham group (Figure 3D), which was validated by volcano plot and GSEA analysis (Figure 3E,F). The celastrol‐induced WAT browning was closely associated with the sympathetic function (Figure 3G–I). Moreover, we identified that Slc27a2 (Fatp2) may be a mediator of WAT browning‐inducing effects of celastrol (Figure 3J). Intriguingly, human studies also revealed that Fatp2 is involved in norepinephrine‐stimulated mitochondrial thermogenesis, and fatty acid transport and utilization.^4^, ^5^, ^6^ Celastrol promoted Fatp2 expression, which was dampened by denervation (Figure 3K,L). Fatp2 expression level in human abdominal subcutaneous adipose tissue is negatively correlated with a body‐mass index (Figure S4A). Isoproterenol increased the Fatp2 expression level in 3T3‐L1 preadipocytes (Figure S4B). The weight‐reducing and WAT browning‐inducing effects of celastrol were dampened by Fatp2 knockdown in iWAT (Figure S4C–G,I–L). In addition, elevated EE and decreased RER induced by celastrol were diminished by Fatp2 knockdown in iWAT (Figure S4M,N). The food intake and motor activity were not affected by Fatp2 knockdown in iWAT (Figure S4H,O). Furthermore, the weight‐reducing and WAT browning‐inducing effects of celastrol were mitigated in Fatp2‐KO mice (Figure 4A–D,F–H,J and K). No differences in food intake and motor activity were observed in Fatp2‐KO mice treated with celastrol, as compared with controls (Figure 4E,L).

**FIGURE 3:**
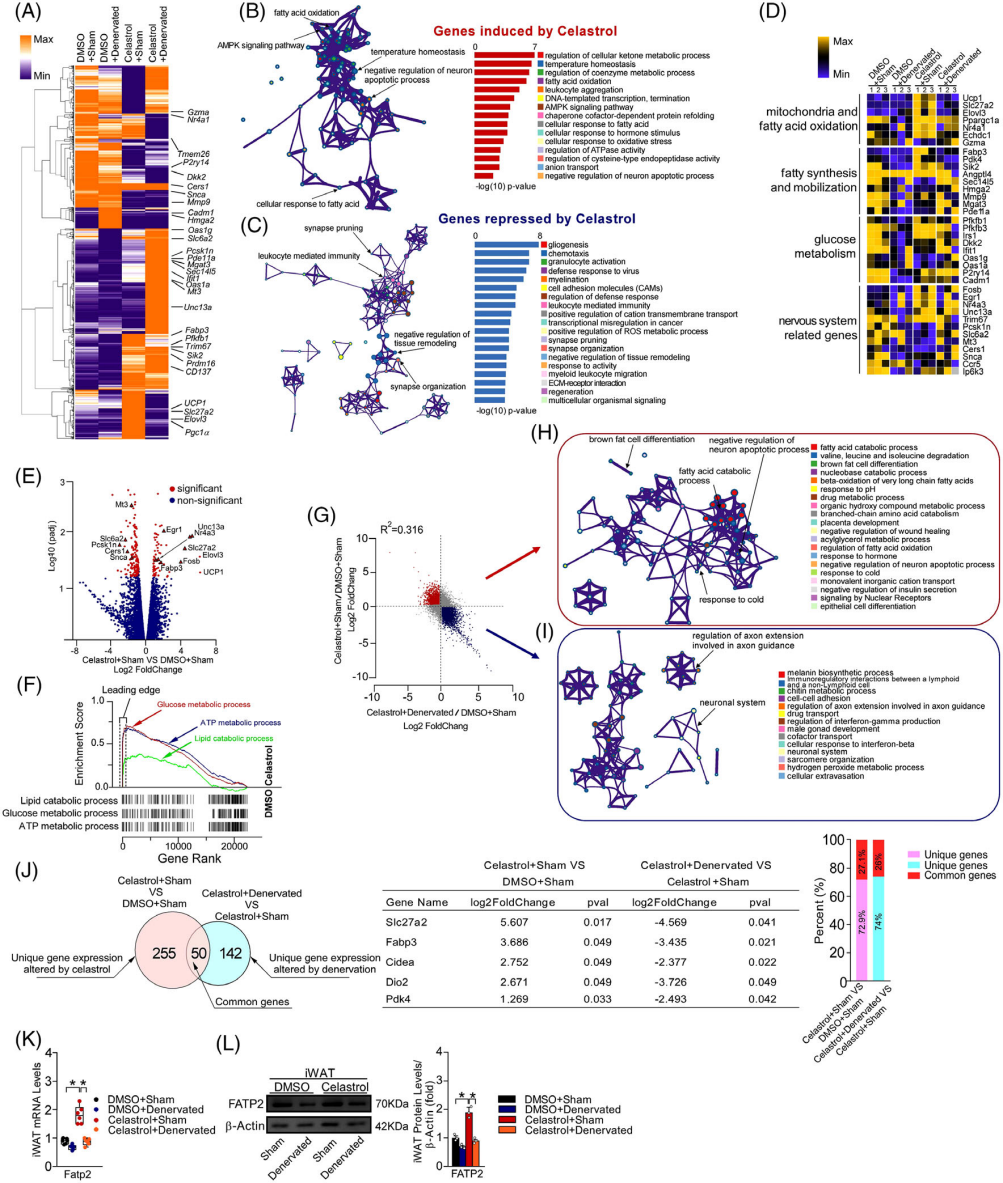
Fatp2 could be a mediator of Celastrol's action. (A) Heatmap depicting differentially expressed genes in inguinal white adipose tissue (iWAT). The browning‐associated genes in iWAT of dimethyl sulfoxide (DMSO) + Sham, DMSO + Denervated, Celastrol + Sham and Celastrol + Denervated groups are indicated in the heatmap labels (*n* = 3 per group). (B,C) Gene Ontology enrichment was based on DEGs that have a *p*‐value smaller than .05. Enrichment analysis for Gene Ontology terms among the genes of a gene–trait correlation module was performed using Metascape. (D) Expression profiles of differentially expressed genes specifically involved in mitochondria and fatty acid oxidation, fat synthesis and mobilization, glucose metabolism and nervous system in iWAT of DMSO + Sham, DMSO + Denervated, Celastrol + Sham and Celastrol + Denervated groups (*n* = 3 per group). (E) Volcano plot showing the differentially affected gene log2‐fold change (Celastrol + Sham / DMSO + Sham) plotted against the −log10 *p*_adj_‐value highlighting significantly regulated genes (red, *p* < .05, *n* = 3 per group, moderated *t*‐test). (F) GSEA shows that the gene sets related to metabolism were significantly upregulated by celastrol. (G) Scatter plot of gene expression values revealing a significant correlation (Pearson's *R* = .316; Spearman's *R* = –.562) between two datasets. (H,I) Correlated genesets from the transcriptome comparison underwent Gene Ontology enrichment analysis to reveal upregulated or downregulated biological processes. (J) Venn diagram of overlapping significantly changed genes (±1.2‐fold, *p* < .05) in the comparisons between two datasets. The top five overlapping genes are presented. (K) Relative mRNA expression levels of Fatp2 (*n* = 6 per group). (L) Representative immunoblots of Fatp2 and β‐Actin in iWAT. Cropped blot images are shown, see Figure S5 for full immunoblots. Quantification of Fatp2 levels (*n* = 6 per group). Data are mean ± standard error of the mean (SEM). *p*‐Values were determined by two‐way analysis of variance (ANOVA) followed by Tukey's multiple comparisons test. *p < .05.

**FIGURE 4:**
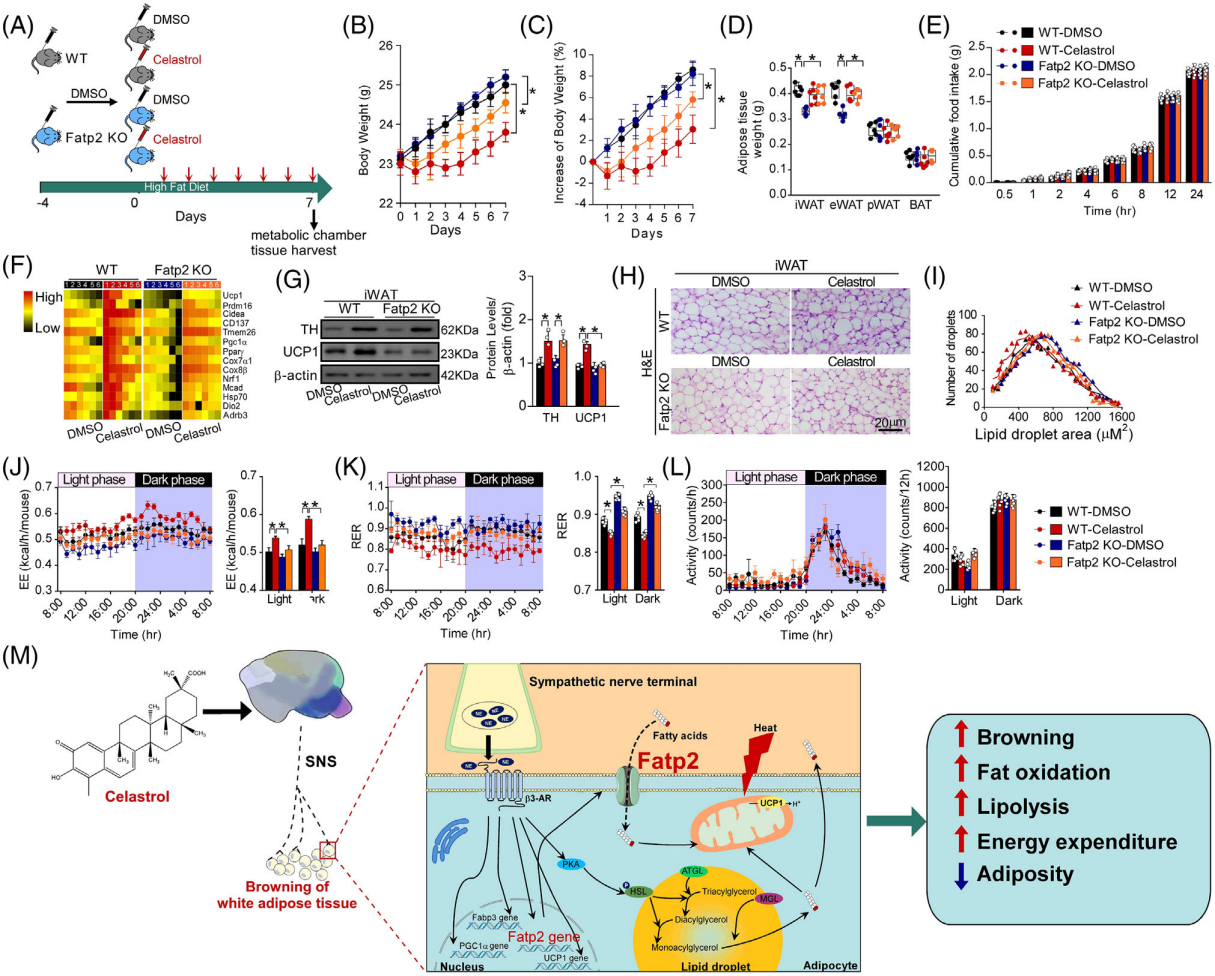
Knockout of Fatp2 abrogates Celastrol‐induced white adipose tissue (WAT) browning. (A) Schematic illustration of experiments. Mice received Celastrol or DMSO once daily for 7 consecutive days after 4‐day acclimation. Mice were kept on high‐fat diet (HFD). Tissues were harvested for molecular analyses on day 8 after the last administration. (B) Body weight (*n* = 15 per group). (C) Percentage change in body weight (*n* = 15 per group). (D) Fat‐pad weight (*n* = 5 per group). (E) Cumulative food intake (*n* = 6 per group). (F) Heatmap shows mRNA levels of browning associated genes in inguinal WAT (iWAT) (*n* = 6 per group). (G) Representative immunoblots of tyrosine hydroxylase (TH), UCP1 and β‐Actin from iWAT, and the quantified ratio of TH/β‐Actin, UCP1/β‐Actin (*n* = 9 per group). Cropped blot images are shown, see Figure S5 for full immunoblots. Values represent mean ± standard error of the mean (SEM). *p‐*Values were determined by two‐way analysis of variance (ANOVA) followed by Tukey's multiple comparisons test. *p < .05, **p < .01 and ***p < .001. (H) Representative images of H&E staining of iWAT (*n* = 9 per group). Scale bar indicates 20 μm. (I) The cell size profiling of adipocytes from iWAT and the quantitative analysis by ImageJ program (*n* = 9 per group). (J) Energy expenditure. Analysis of covariance (ANCOVA) was used to determine statistical differences for in vivo metabolic analyses. (K) Respiratory exchange ratio (RER). (L) Ambulatory physical activity (*n* = 5 per group). Values represent mean ± SEM. *p‐*Values were determined by non‐paired two‐tailed Student's *t*‐test. *p < .05. (M) Schematic summary of the mechanism underlying the browning‐inducing effects of Celastrol

In conclusion, as shown in Figure 4M, our findings suggest that celastrol activates the sympathetic‐adiposity axis to promote WAT browning and weight loss. Fatp2 may play essential roles in mediating celastrol‐induced WAT browning. This study may provide a novel strategy for the treatment of obesity.

## FUNDING

This work was supported by grants from the National Natural Science Foundation of China (No. 81471064 and No. 81670779 and 81870590; R.Z.), the National Key Research and Development Program of China (2017YFC1700402; R.Z.), the Beijing Municipal Natural Science Foundation (No. 7162097 and No. H2018206641; R.Z.), the Peking University Research Foundation (No. BMU20140366; R.Z.), and the Peking University People's Hospital Research and Development Funds (No. RYD2019‐01; Z.H.).

## AUTHOR CONTRIBUTIONS

B.W. and X.Y. performed the experiment, analyzed the data, made the figures, and wrote the paper. M.Z., Z.S., Z.H., C.Z., B.G. and J.L. participated in experiments. L.Q. and W.Z. edited the manuscript. R.Z. conceived the study, designed experiments, and wrote and edited the paper. All authors reviewed and approved the manuscript for submission.

## DATA AND MATERIALS AVAILABILITY

All data associated with this study are present in the paper or Supplementary Materials.

## CONFLICT OF INTEREST

The authors declare no conflict of interest.

## Supporting information



Supporting information

FigureS1

FigureS2

FigureS3

FigureS4

FigureS5

FigureS6
