# Supplementary material for: Celastrol prevents high‐fat diet‐induced obesity by promoting white adipose tissue browning
Source: Clin Transl Med. 2021 Dec 15;11(12):e641. doi: 10.1002/ctm2.641 (PMC8673360; doi:10.1002/ctm2.641)
Supplement: Supplementary file 1 — Supporting information [file CTM2-11-e641-s003.docx]

**Supplementary Materials for**

**Celastrol prevents high-fat diet-induced obesity**

**by promoting white adipose tissue browning**

**(CTM2-2021-07-1416.R1)**

**Materials and Methods**

**Reagent**

| **REAGENT** | **SOURCE** | **IDENTIFIER** |
| --- | --- | --- |
| Rabbit polyclonal to UCP1 (1:2000) | Abcam | ab10983 |
| Rabbit polyclonal to PGC1α (1:1000) | Santa Cruz Biotechnology | sc-13067 |
| Rabbit polyclonal to TH (1:5000) | Millipore | AB152 |
| Rabbit polyclonal to FATP2 (1:2000) | ThermoFisher Scientific | PA5-30420 |
| Rabbit polyclonal to FATP2 (1:2000) | Bioss | bs-3936R |
| Mouse monoclonal to FATP2 (1:500) | Santa Cruz Biotechnology | sc-393906 |
| Mouse monoclonal to β-Actin (1:10000) | Sigmaaldrich | A5316 |
| Rabbit polyclonal to α/β-Tubulin (1:10000) | Cell Signaling Technology | 2148S |
| Anti-rabbit IgG/Alexa Fluor 488 (1:500) | Bioss | bs-02950-AF488 |
| HRP AffiniPure Goat Anti-Rat IgG(H+L) (1:5000) | Biodragon | BF03008 |
| HRP AffiniPure Goat Anti-Mouse IgG(H+L) (1:5000) | Biodragon | BF03001 |
| Celastrol | BOC Science Company | 34157-83-0 |
| 6-hydroxydopamine (6-OHDA) | Sigma-Aldrich | H4381 |
| TranZol up | TransGen Biotech | ET111-01 |
| TransScript one-step gDNA removel and cDNA synthesis Super MiX | TransGen Biotech | AT311-03 |
| TransStart Top Green qPCR Super Mix | TransGen Biotech | AQ141-04 |
| Protease Inhibitor Cocktail | Bimake | B14002 |
| Phosphatase Inhibitor Cocktail | Bimake | B15002 |

**Mice**

C57BL/6 mice were purchased from Charles River Laboratories Beijing Branch (Beijing Vital River Laboratory Animal Technology Co., Ltd.) and the Department of Laboratory Animal Science of Peking University Health Science Center. The Slc27a2^-/-^ (Gene ID: 26458) mouse line was produced by Dr. Ligong Chen’s laboratory (Tsinghua University) using the CRISPR/Cas9 technology. The Adrb3 KO (JAX 006402) mouse was kindly provided by Dr. Wenwen Zeng (Tsinghua University). Male mice were used for all the experiments described in this study. 60 kcal% High Fat Diet (HFD, Research Diets, D12492i, Frozen storage: -80 °C) was applied to 8-week-old mice. HFD was replaced every day to keep fresh. We maintained mice on a 12 h light-dark cycle in a temperature-controlled high barrier facility with unrestricted access to food and water. Thermoneutral exposures were performed at 30^o^C in a light- and humidity-controlled climatic chamber at Animal Facility of Peking University Health Science Center. All animal experiments were performed in compliance with institutional guidelines and had been approved by PKU Institutional Animal Care and Use Committee (Protocol Registry Number: LA2019340). During all procedures of experiments, the number of animals and their suffering by treatments were minimized.

**Ethical considerations**

This study was approved by the Ethical Committee of Peking University People's Hospital (2019PHB205-01). Written informed consent was obtained from all individual participants included in the study. If patients were unable to give written consent, their contact person gave the consent. All data were kept confidential and processed anonymously.

**Subjects**

The study included 31 human subcutaneous adipose tissue samples from 5 obese patients (BMI≥30 kg/m^2^) and 26 age- and sex-matched nonobese subjects (BMI ＜30 kg/m^2^), collected during bariatric surgery or abdominal surgical procedure for benign disease. The subjects were all apparently healthy, aside from the surgery indication, with no history of alcohol overconsumption. No patient had overt diabetes or lipodystrophia, and no one was under antihypertensive treatment with β-blockers. Weight was measured before surgery.

**Administration of celastrol**

For intraperitoneal (i.p.) treatment, mice received 25 μl of DMSO for four days as acclimation before celastrol or DMSO treatment. Celastrol was dissolved and administered to mice in DMSO (25 μl) solution. Vehicle groups received 25 μl of DMSO for control purposes. All treatments were performed within 90 minutes before dark cycle and mice received celastrol or DMSO daily for 7 days.

For long-term (two months) treatment of celastrol, mice received 25 μl of DMSO (i.p.) for four days as acclimation before celastrol or DMSO treatment. Mice treated with celastrol at dose levels of 1000 μg/kg, 100 μg/kg, 10 μg/kg and 1 μg/kg. Vehicle groups received 25 μl of DMSO for control purposes. The celastrol treatment at doses of 1,000 μg/kg, 100 μg/kg resulted in a severe decline in food-intake and body weight in the HFD mice by 4-5 days, and thus the experiments were terminated for animal welfare considerations.

**Food intake and body weight measurements**

Food intake and body weight were monitored daily and percent increase of body weight was calculated by following equation: 100 X (body weight after injection of celastrol-body weight of initial)/ body weight of initial.

**Metabolic chamber**

At the day 6 of celastrol treatment, we placed the mice in metabolic chambers with fresh food and water provided every day to acclimate for 24 hr. The day 7, we administered celastrol (1 μg/kg) or DMSO within 90 minutes before dark cycle and then monitored the oxygen consumption (VO^2^), carbon dioxide production (VCO^2^), respiratory-exchange-ratio (RER), energy expenditure (EE) and activity for 24 hr (LE1305 Physiocage 00; LE405 O_2_/CO_2_ Analyzer; LE400 Air Supply and Swithching). Metabolism v2.2.01 was used to analyze the data. These metabolic parameters were adjusted for mouse adiposity.

**Sympathetic denervation of iWAT**

8-week-old mice received 20 microinjections of 6-hydroxydopamine [6-OHDA (Sigma); 1 μl per injection, 9 mg/ml in 0.15 M NaCl containing 1% (w/v) ascorbic acid] throughout the right or both inguinal fat pads. Sham operated fat pads received an equal volume of vehicle. Body weights were monitored throughout the duration of the experiment. Two weeks (unilateral) or five weeks (bilateral) after 6-OHDA injections, mice were treated with 1 μg/kg celastrol daily for a week, then the iWAT and BAT were harvested for histological/ immunofluorescence assessment or processed for qPCR. In this study, no cardiovascular or renal toxicity was observed.

**Total protein extraction and western blotting**

To prepare total lysates from the fat pads, samples were lysed in ice-cold tissue RIPA buffer [1% Trinton X 100; 10 mM Na_2_HPO_4_ (Sodium phosphate); 150 mM NaCl (Sodium chloride); 1% DOC (Sodium deoxycholate or deoxycholic acid); 5 mM EDTA; 5 mM NaF (sodium Fluoride); 0.1% SDS]. Following five minutes of homogenization, lysates were centrifuged at 12000 r for 15 minutes at 4°C. Supernatants from the fat pads were used as protein extracts. The concentration of each sample was calculated by BCA method and equal amount of protein from each sample was denatured by boiling at 100ºC for 5 minutes. Proteins were separated on sodium dodecyl sulfate poly acrylamide (SDS-PAGE) gels, and transferred onto polyvinylidene fluoride (PVDF) membrane at 4°C, 300 mA for two hours. The membranes were blocked for two hours in 5% skimmed milk. The membranes were then incubated with primary antibody in 5% BSA-TSBT at 4°C. After overnight incubation, the membrane was washed three times in TBST for 15 minutes, followed by incubation with secondary antibody in TBST with 5% blocking reagent for two hours at room temperature. Following three cycles of 15-minute washes with TBST, the membranes were developed using a chemiluminescence assay.

**Hematoxylin and eosin (H&E) staining**

The WATs and BAT were collected from the mice treated with celastrol (1 μg/kg) or DMSO for a week. Fats were immediately dissected and fixed in 4% paraformaldehyde solution for 48 hr followed by cryopreservation in 25% sucrose solution (wt/vol) overnight and subsequent freezing in OCT compound (Tissue-Tek). We stored samples in optimal cutting temperature compound (OCT) for frozen. Samples were sectioned, and H&E stained. The cell size was calculated by Image J.

**Immunofluorescence**

For iWAT, eWAT, BAT and hypothalamus immunofluorescence, animals were killed and fats were immediately dissected and fixed in 4% paraformaldehyde solution for 48 hr. Tissues were embedded in OCT and 10 μm sections of the entire block prepared according to standard procedures. Frozen Section of the tissue was used to detect TH by immunofluorescence. Sections were blocked with 10% (v/v) normal horse serum in 1X phosphate buffer and incubated overnight (4℃) with anti-TH (1:200). After washing with PBS 3 X 15min, sections were incubated for 2hr at room temperature with Alexa-Fluor 488-conjugated secondary antibodies (1:200) and nuclei were stained with 4’,6-diamidino-2-phenylindole (DAPI). Stained slides were analyzed using a microscope (Olympus) at the indicated magnification and images captured by a digital camera.

**Quantitative real-time PCR**

Total RNA for quantitative real-time PCR (qPCR) was extracted from tissues with TRIzol. The RNA quality and quantity were determined using a NanoDrop 5500 (Thermo). The total RNA was used for mRNA-Sequencing or qPCR. Total RNA (1μg) was reverse transcribed to complementary DNA (cDNA) using First Strand cDNA Synthesis Kit, according to the manufacturers' instructions. The relative expression of mRNAs was determined by qPCR using the SYBR Green PCR system (BioRad). The relative expression of genes of interest was calculated by comparative Ct method and GAPDH was used as an endogenous control.

**Sample Preparation for LC–MS Analysis**

Samples for LC–MS analysis were extracted from snap-frozen tissues. In brief, in order to avoid tissue heterogeneity, the entire organs were cut on dry ice and mixed thoroughly. Only 50 mg snap-frozen tissues were weighed and homogenized using superfine homogenizer in 500 μl of chloroform/methanol (v:v = 2:1) containing 1 μg internal standards PG (14:0/14:0) and BMP (14:0/14:0). Aqueous solution (125 μl) was added to the mixture of chloroform/methanol for liquid-liquid extraction. Samples were vortexed for 1 min and vortexing was repeated three times. After low speed centrifugation at 1000 rpm, the lower chloroform layer was transferred and dried under nitrogen. Dried pellets were stored in −80 °C freezer.

**LC–MS Analysis**

For fatty acid analysis, samples were re-suspended in 100 uL of dichloromethane (CH2Cl2)/ Methanol (MeOH) (v: v =1:1). The UPLC system was coupled to a Q-Exactive orbitrap mass spectrometer (Thermo Fisher, CA) equipped with a heated electrospray ionization (HESI) probe. Lipid extracts were separated by a CORTECS C18 (100 × 2.1 mm 1.6 μm) column (Waters, USA). A binary solvent system was used, in which mobile phase A consisted of ACN:H_2_O (60:40), 10 mM Ammonium acetate, and mobile phase B of IPA:ACN (90:10). A 18-minute gradient with flow rate of 220 μl/min was used. Linear gradient was as follows: 0 min, 30% B; 2.5 min,30% B;8 min 50% B; 10 min, 98% B; 15 min 98% B; 15.1 min,30% B; 18 min 30% B. Column chamber and sample tray were held at 40°C and 10°C, respectively. Data with mass ranges of m/z 150-2000 was acquired at negative ion mode. The full scan was collected with resolution of 70,000. The source parameters are as follows: spray voltage: 3000v; capillary temperature: 320^o^C; heater temperature: 300^o^C; sheath gas flow rate: 35 Arb; auxiliary gas flow rate: 10 Arb. Data analysis and lipid identification were performed by the tracefinder (Thermo Fisher, CA) according to endogenous MS database by accurate masses.

**Mouse studies for β-blocker**

For β-blocker experiments, propranolol (10 mg/kg/day, TOCRIS Bioscience-0624) and SR59230A (2.5 mg/kg/day, TOCRIS Bioscience-1511) were administered by i.p. injection daily at 6 pm.

**RNA-sequencing**

A total amount of 3 μg RNA per sample was used as input material for the RNA sample preparations. Sequencing libraries were generated using NEBNext® Ultra™ RNA Library Prep Kit for Illumina® (NEB, USA) following manufacturer’s recommendations and index codes were added to attribute sequences to each sample. Briefly, mRNA was purified from total RNA using poly-T oligo-attached magnetic beads. Fragmentation was carried out using divalent cations under elevated temperature in NEBNext First Strand Synthesis Reaction Buffer (5X). First strand cDNA was synthesized using random hexamer primer and M-MuLV Reverse Transcriptase (RNase H). Second strand cDNA synthesis was subsequently performed using DNA Polymerase I and RNase H. Remaining overhangs were converted into blunt ends via exonuclease/polymerase activities. After adenylation of 3’ ends of DNA fragments, NEBNext Adaptor with hairpin loop structure were ligated to prepare for hybridization. In order to select cDNA fragments of preferentially 150~200 bp in length, the library fragments were purified with AMPure XP system (Beckman Coulter, Beverly, USA). Then 3 μl USER Enzyme (NEB, USA) was used with size-selected, adaptor-ligated cDNA at 37°C for 15 min followed by 5 min at 95 °C before PCR. Then PCR was performed with Phusion High-Fidelity DNA polymerase, Universal PCR primers and Index (X) Primer. At last, PCR products were purified (AMPure XP system) and library quality was assessed on the Agilent Bioanalyzer 2100 system.

The clustering of the index-coded samples was performed on a cBot Cluster Generation System using TruSeq PE Cluster Kit v3-cBot-HS (Illumia) according to the manufacturer’s instructions. After cluster generation, the library preparations were sequenced on an Illumina Hiseq 2000/2500 platform and 100 bp/50bp single-end reads were generated.

Differential expression analysis of two conditions/groups (two biological replicates per condition) was performed using the DESeq R package (1.10.1). DESeq provide statistical routines for determining differential expression in digital gene expression data using a model based on the negative binomial distribution. The resulting P-values were adjusted using the Benjamini and Hochberg’s approach for controlling the false discovery rate. Genes with an adjusted P-value <0.05 found by DESeq were assigned as differentially expressed.

Gene Ontology (GO) enrichment analysis of differentially expressed genes was implemented by the GOseq R package, in which gene length bias was corrected. GO terms with corrected P value less than 0.05 were considered significantly enriched by differential expressed genes.

**Cell culture**

3T3-L1 cells were cultured to confluence in Dulbecco’s modified Eagle’s medium (DMEM, Thermo) containing 10% (vol/vol) fetal bovine serum (FBS, Biological Industries), with the medium changed every 2 d at 37 °C in a 5 % CO_2_ incubator. At 2 d after cell confluence, differentiation was initiated by adding differentiation medium 1 [0.5 Mm 3-isobutyl-1-methylxanthine (IBMX), 0.25 μM dexamethasone, 1 μg/mL insulin in DMEM containing 10% (vol/vol) FBS].

**RNAi-mediated gene knockdown**

All siRNAs and scramble siRNA (Ctrl) were purchased from Ribobio. The targeting sequences for siRNA were 5'-GGTATGAGCTGATCAAGTA-3' (Fatp2). siRNAs were transfected onto the differentiated 3T3-L1 cells with Lipofectamin-2000 reagents (Invitrogen). At 24 h after infection, cells were treated with 10 nM isoproterenol or vehicle as indicated. After 24 h, cells were harvested, and mRNA was isolated for qPCR analysis.

**Adeno-associated viruses preparation and injection**

Mice were anesthetized by isofluorane and placed in a prone position. The skin above the inguinal fat pad was prepared in a sterile fashion and an incision was performed to expose the inguinal fat pad. Virus diluted in sterile PBS: 2.0×10^10^vg/20μl for each mouse was injected at multiple sites of the inguinal fat pad using a 0.3cc, 31G insulin syringe. After the injection, incisions were closed with surgical clips.

**Statistical analysis**

Statistical significance was determined with SPSS (Windows version 26) or GraphPad Prism software (version 7.0; Origin Laboratory). Student’s t test was used to detect differences within groups when applicable (2-tailed). One-way ANOVA (ANOVA) was used to compare differences among all groups, and Bonferroni post hoc testing was used to detect differences among mean values of the groups. ANCOVA were used to determine statistical differences for in vivo metabolic analyses. Sample sizes, statistical tests and p values are indicated in the text, figures, and figure legends. A p value less than 0.05 was considered statistically significant. Data are presented as the mean ± SEM. * p < 0.05, ** p < 0.01, *** p < 0.001.

**Supplementary table 1:** Primers used in this study.

| **Primer** | **Forward Primer 5'-3'** | **Reverse Primer 5'-3'** |
| --- | --- | --- |
| Ucp1 | ACTGCCACACCTCCAGTCATT | CTTTGCCTCACTCAGGATTGG |
| Prdm16 | CAGCACGGTGAAGCCATTC | GCGTGCATCCGCTTGTG |
| Cidea | TGCTCTTCTGTATCGCCCAGT | GCCGTGTTAAGGAATCTGCTG |
| CD137 | CGTGCAGAACTCCTGTGATAAC | GTCCACCTATGCTGGAGAAGG |
| Tmem26 | ACCCTGTCATCCCACAGAG | TGTTTGGTGGAGTCCTAAGGTC |
| Tbx1 | GTCAAGGCTCCGGTGAAGAAG | GCTGATTGAACTCGTCCCACA |
| Pgc1α | AGCCGTGACCACTGACAACGAG | GCTGCATGGTTCTGAGTGCTAAG |
| Pparα | GGGTACCACTACGGAGTTCACG | CAGACAGGCACTTGTGAAAACG |
| Pparγ | GTGCCAGTTTCGATCCGTAGA | GGCCAGCATCGTGTAGATGA |
| Cox7α1 | CAGCGTCATGGTCAGTCTGT | AGAAAACCGTGTGGCAGAGA |
| Cox8β | GAACCATGAAGCCAACGACT | GCGAAGTTCACAGTGGTTCC |
| Nrf1 | CAGCAACCCTGATGGCACCGTGTC | GGCCTCTGATGCTTGCGTCGTCTG |
| Mcad | ATGACGGAGCAGCCAATGAT | TCGTCACCCTTCTTCTCTGCTT |
| HSP70 | TGGTGCTGACGAAGATGAAG | AGGTCGAAGATGAGCACGTT |
| Dio2 | AGAGTGGAGGCGCATGCT | GGCATCTAGGAGGAAGCTGTTC |
| Adrb3 | TTGTCCTGGTGTGGATCGTG | TTGGAGGCAAAGGAACAGCA |
| Adrb2 | ATGTCGGTTATCGTCCTGGC | GGTTTGTAGTCGCTCGAACTTG |
| Adrb1 | CTCATCGTGGTGGGTAACGTG | ACACACAGCACATCTACCGAA |
| PGP 9.5 | AGGGACAGGAAGTTAGCCCTA | AGCTTCTCCGTTTCAGACAGA |
| DβH | GTGGTCCTCGATCCCGAAG | CTCTCTGTCTGCGTTTGTGAA |
| Fatp2 | CGAGACGAGACGCTCACCTA | ACGAATGTTGTAGTTGAGGCAC |
| β-Actin | GTATGCCTCGGTCGTACCA | CTTCTGCATCCTGTCAGCAA |

**SUPPLEMENTAL LEGENDS**

FIGURE S1 Celastrol prevents HFD-induced obesity. (A) Schematic illustration of experiments. Mice treated daily with celastrol (1,000 μg/kg, 100 μg/kg, 10 μg/kg, and 1 μg/kg) or DMSO by i.p. injection for 8 weeks kept on HFD; adipose tissues were collected after the final treatment (n = 9 per group). (B) Body weight changes were compared among mice treated daily with celastrol (1,000 μg/kg, 100 μg/kg, 10 μg/kg, and 1 μg/kg) or DMSO by i.p. injection for 8 weeks kept on HFD (n = 9 per group). (C) Food intake in the mice fed a HFD treated daily with celastrol (1,000 μg/kg, 100 μg/kg, 10 μg/kg, and 1 μg/kg) or DMSO by i.p. injection for 8 weeks (n = 9 per group). (D) Cumulative food intake in the mice fed a HFD treated daily with celastrol (10 μg/kg and 1 μg/kg) or DMSO by i.p. injection for 8 weeks (n = 9 per group). (E) Ucp1 gene expression in different iWAT from mice treated with celastrol (10 μg/kg or 1 μg/kg) or DMSO for 1, 2, 4, 8 and 12 weeks kept on HFD (n = 9 per group). (F) Representative immunoblots of UCP1, PGC1α and β-Actin from iWAT, and the quantified ratio of UCP1/β-Actin, PGC1α/β-Actin (n = 9 per group) (see FIGURE S6 for full immunoblots). (G) Representative images of H&E staining of adipocytes from iWAT (n = 9 per group). Scale bar indicates 20 µm. (H) The cell size profiling of adipocytes from iWAT and the quantitative analysis by ImageJ program (n = 8-9 per group). (I) Relative mRNA expression of browning markers in iWAT of mice treated daily with celastrol (0.1 μg/kg) or DMSO by i.p. injection for 7 days kept on HFD, adipose tissues were collected 8 h after the final treatment (n = 6 per group). Values represent mean ± SEM. P values were determined by non-paired two-tailed Student’s t test. *P < 0.05, **P < 0.01.

FIGURE S2 Celastrol-promoted browning is SNS-dependent. (A) The regulatory mechanism of hypothalamus on WAT browning. (B) Representative immunoblots of TH and β-Actin from iWAT, and the quantified ratio of TH/A-actin (n = 9 per group). Representative blot images are shown, see FIGURE S5 for full immunoblots. Values represent mean ± SEM. P values were determined by two-way ANOVA followed by Tukey’s multiple comparisons test. *P < 0.05, **P < 0.01. (C) Representative immunofluorescence images of TH in iWAT (n = 8 per group). Scale bar indicates 20 μm. (D) Gene set enrichment analysis (GSEA) shows that the gene sets related to nerve activity were significantly upregulated by celastrol treatment.

FIGURE S3 Celastrol-promoted browning is β-adrenoreceptor-dependent. (A) Diagram of experimental design. β-blocker (propranolol, 10 mg/kg/day; SR59230A, 2.5 mg/kg/day) or DMSO were injected (i.p.) for 7 consecutive days starting at day 0 after acclimation (4 days), mice intraperitoneally (i.p.) received celastrol (1 μg/kg) or DMSO daily for 7 days, and tissues were harvested for molecular analyses at day 8 after the last administration. The arrow indicates the time of celastrol or DMSO injection. (B) Body weight (n = 6 per group). (C) Percentage change in body weight (n = 6 per group). (D) Cumulative food intake (n = 6 per group). (E) Heatmap shows mRNA levels of browning-associated genes in iWAT (n = 6 per group). (F) Representative immunoblots of TH, UCP1, PGC1α and β-Actin from iWAT, and the quantified ratio of TH/β-Actin, UCP1/β-Actin, and PGC1α/β-Actin (n = 6 per group). Cropped blot images are shown, see FIGURE S6 for full immunoblots. (G) Representative images of H&E staining of iWAT (n = 6 per group). Scale bar indicates 20 μm. (H) The cell size profiling of adipocytes from iWAT and the quantitative analysis by ImageJ program (n = 6 per group). Values represent mean ± SEM. P values were determined by two-way ANOVA followed by Tukey’s post hoc test. *P < 0.05 and **P < 0.01.

FIGURE S4 Knockdown of Fatp2 diminishes celastrol-induced WAT browning. (A) Correlation between Fatp2 expression in abdominal SAT and BMI. Fatp2 mRNA expression, quantified by qPCR and normalized to β-actin mRNA, was correlated with BMI in 31 patients. Statistical analysis was performed by Pearson correlation. (B) Fatp2 knockdown 3T3-L1 adipocytes were stimulated in the presence or absence of isoproterenol (10 nM) for 2 hr. mRNA levels were measured using qRT-PCR. Data are presented as the mean ± SEM of triplicate wells from a representative experiment. P values were determined by two-way ANOVA followed by Tukey’s multiple comparisons test. *P < 0.05. (C) Construction of the AAV9-shRNA-Fatp2 vector. (D) Diagram of the experimental design. AAV9-shRNA-Fatp2 or AAV9-shRNA-Ctrl were injected in the iWAT of mice, and after 2 weeks mice were fed a HFD. Celastrol (1 μg/kg) or DMSO were injected (i.p.) for 7 consecutive days starting on day 0 after acclimation, then indirect calorimetry recording was performed at day 6 and tissues were harvested for molecular analyses at day 8 after the last administration. The arrow indicates the time of celastrol or DMSO injection. (E) Body weight (n = 12 per group). (F) Percentage change in body weight (n = 12 per group). (G) Fat-pad weight (n = 12 per group). (H) Cumulative food intake (n = 6 per group). (I) Heatmap shows mRNA levels of browning associated genes in iWAT of celastrol and DMSO treated mice (n = 6 per group). (J) Representative immunoblots of TH, UCP1 and β-Actin from iWAT, and the quantified ratio of TH/β-Actin and UCP1/β-Actin (n = 12 per group). Cropped blot images are shown, see FIGURE S6 for full immunoblots. Values represent mean ± SEM. P values were determined by two-way ANOVA followed by Tukey’s multiple comparisons test. *P < 0.05. (K) Representative images of H&E staining of iWAT, scale bar indicates 20 μm (n = 12 per group). (L) The cell size profiling of adipocytes from iWAT and the quantitative analysis by ImageJ program (n = 12 per group). (M) Energy expenditure. ANCOVA were used to determine statistical differences for in vivo metabolic analyses. (N) Respiratory exchange ratio (RER). (O) Ambulatory physical activity (n = 5 per group). Values represent mean ± SEM. P values were determined by non-paired two-tailed Student’s t test. *P < 0.05.

FIGURE S5 Full immunoblots relating to FIGURE 1, 2, 3, 4 and FIGURE S2.

FIGURE S6 Full immunoblots relating to FIGURE S1, S3 and S4.
