# Supplementary figures and images for: Celastrol prevents high‐fat diet‐induced obesity by promoting white adipose tissue browning
Source: Clin Transl Med. 2021 Dec 15;11(12):e641. doi: 10.1002/ctm2.641 (PMC8673360; doi:10.1002/ctm2.641)

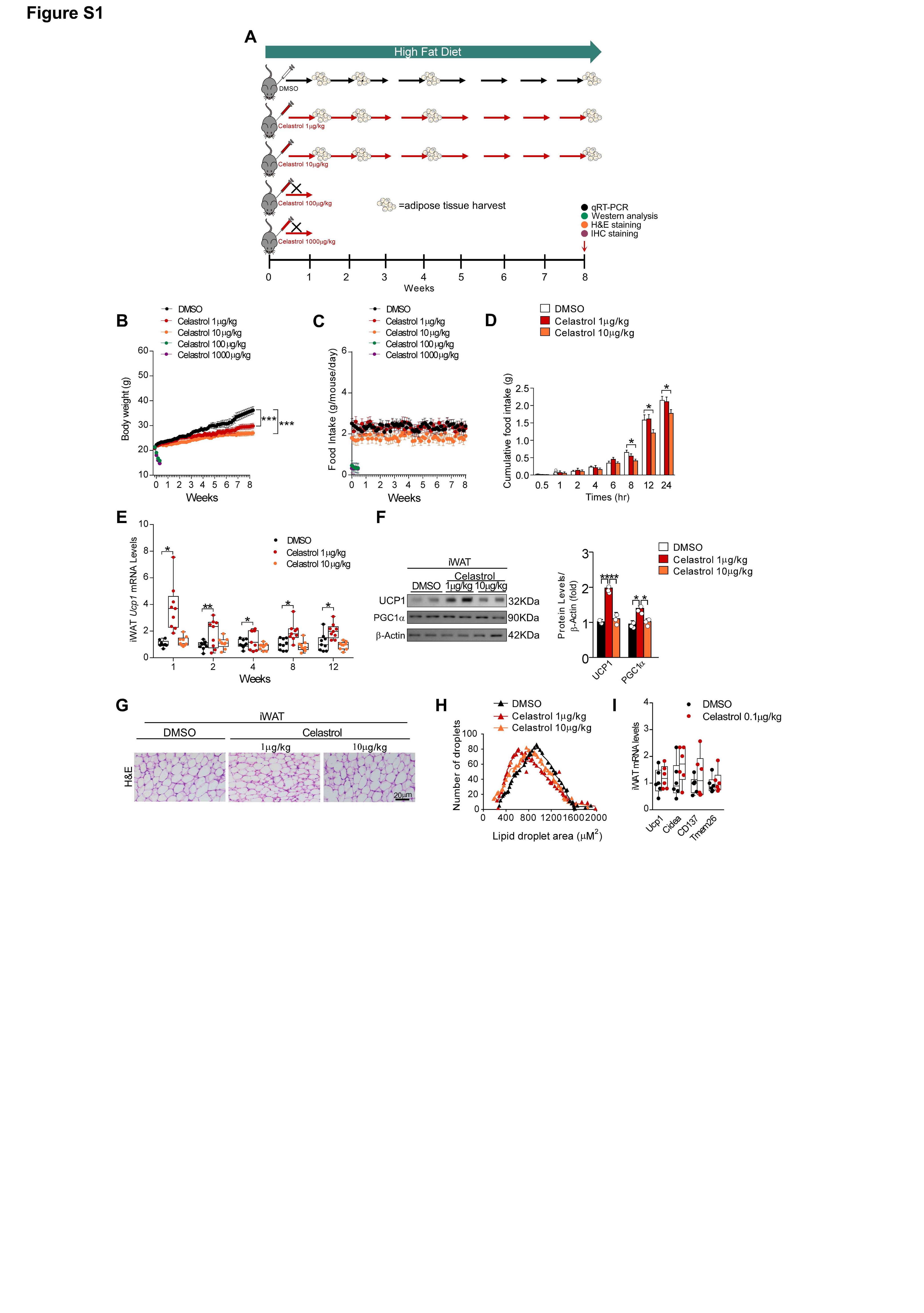

Supplement: Supplementary file 2 — FigureS1 [file CTM2-11-e641-s005.jpg]

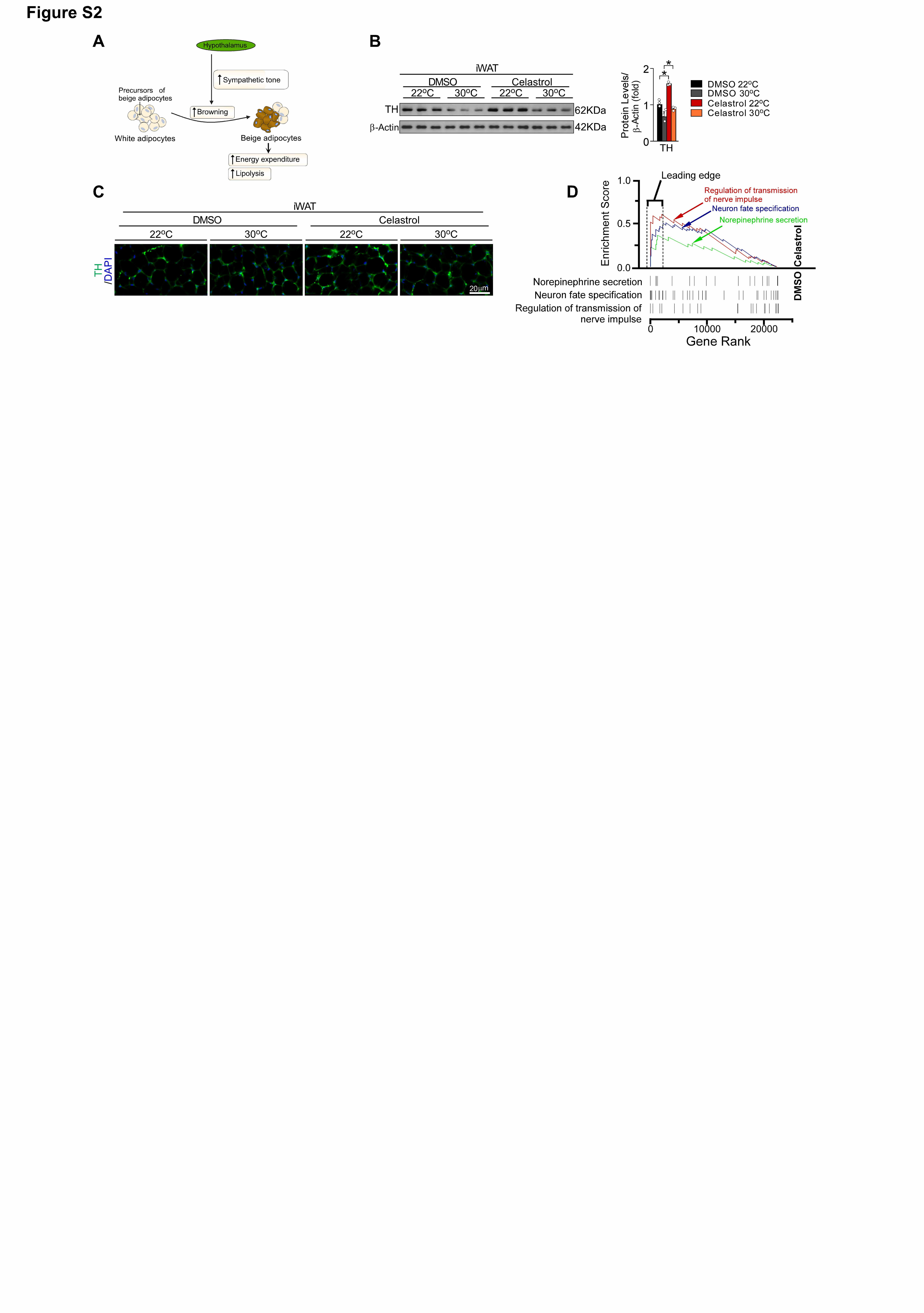

Supplement: Supplementary file 3 — FigureS2 [file CTM2-11-e641-s001.jpg]

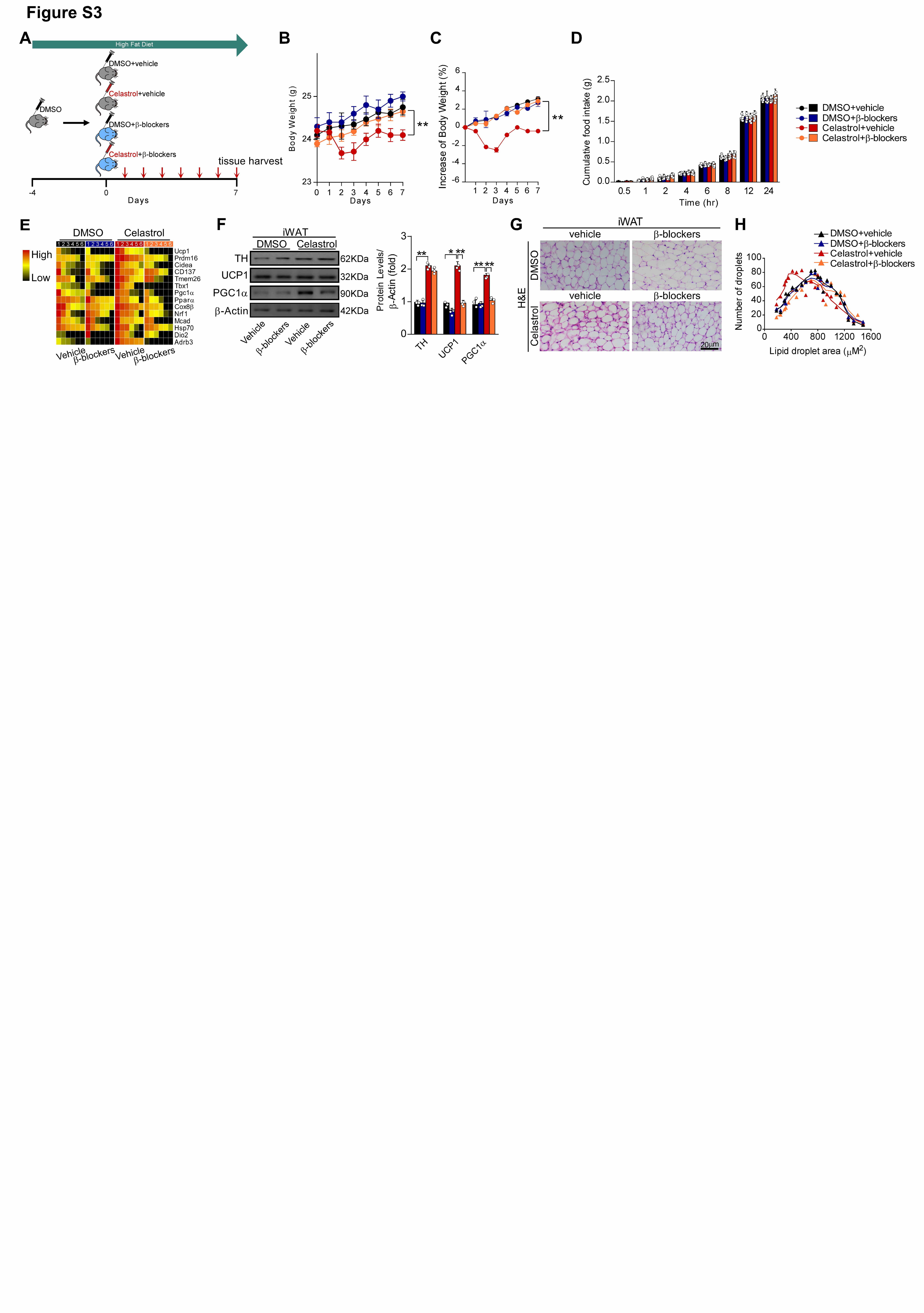

Supplement: Supplementary file 4 — FigureS3 [file CTM2-11-e641-s006.jpg]

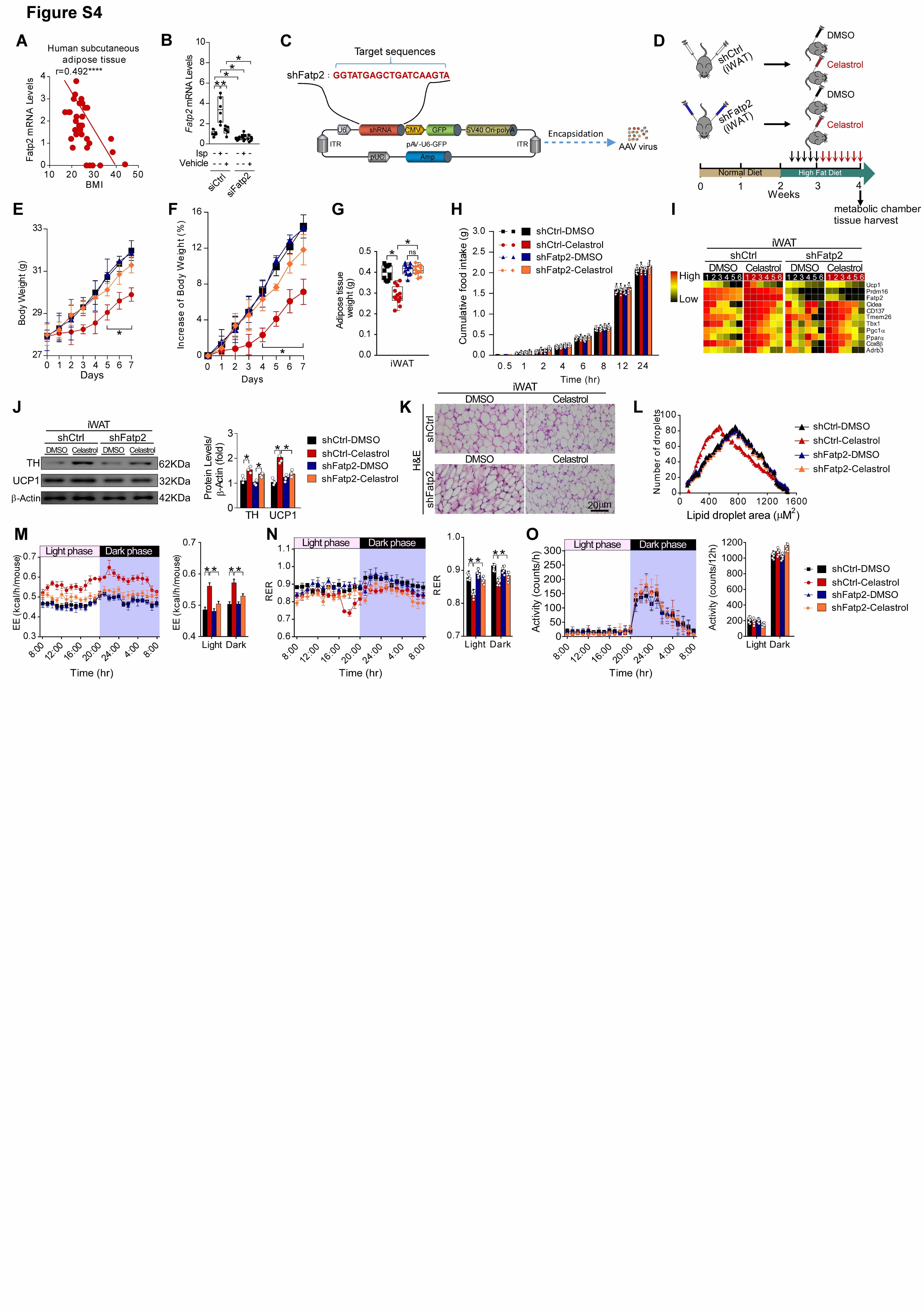

Supplement: Supplementary file 5 — FigureS4 [file CTM2-11-e641-s007.jpg]

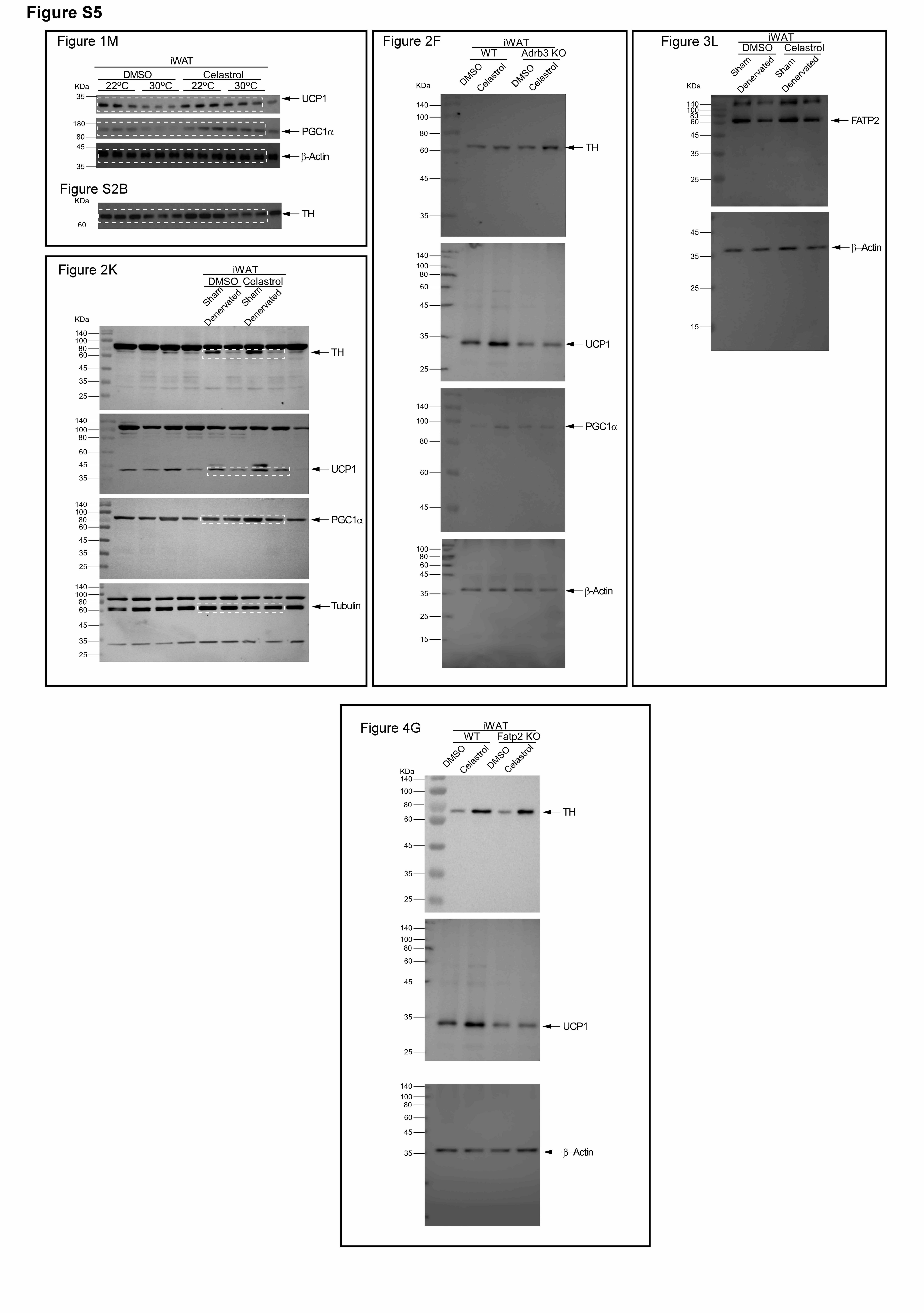

Supplement: Supplementary file 6 — FigureS5 [file CTM2-11-e641-s004.jpg]

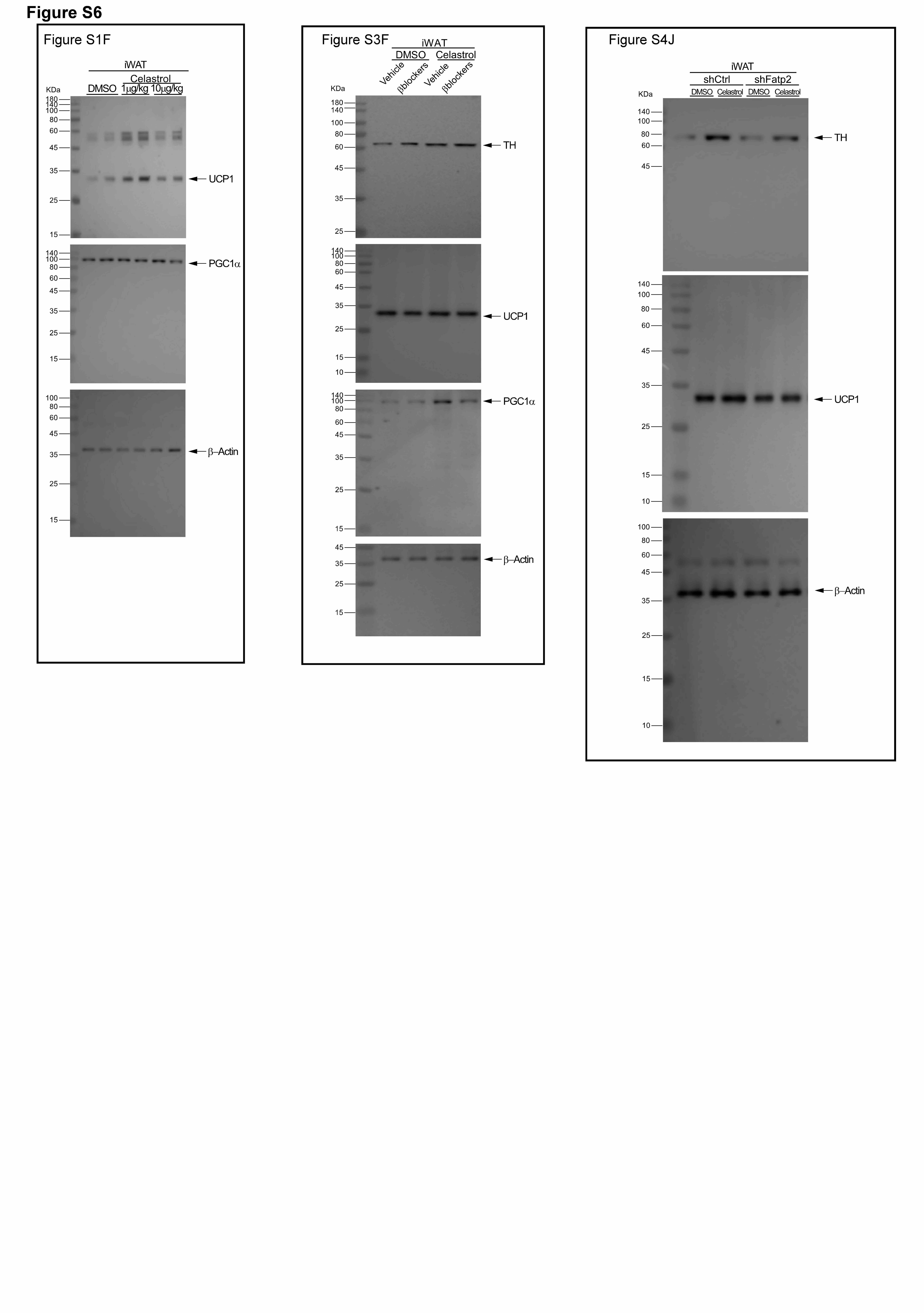

Supplement: Supplementary file 7 — FigureS6 [file CTM2-11-e641-s002.jpg]
